# Clinical Experience of Auditory Brainstem Response Testing on Pediatric Patients in the Operating Room

**DOI:** 10.1155/2012/350437

**Published:** 2011-11-17

**Authors:** Guangwei Zhou, Briana Dornan, Wheaton Hinchion

**Affiliations:** ^1^Department of Otolaryngology and Communication Enhancement, Children's Hospital Boston, 300 Longwood Avenue LO-367 Boston, MA 02115, USA; ^2^Department of Otology and Laryngology, Harvard Medical School, Boston, MA 02115, USA

## Abstract

*Objectives*. To review our experience of conducting auditory brainstem response (ABR) test on children in the operating room and discuss the benefits versus limitations of this practice. *Methods*. Retrospective review study conducted in a pediatric tertiary care facility. A total of 267 patients identified with usable data, including ABR results, medical and surgical notes, and follow-up evaluation. *Results*. Hearing status successfully determined in all patients based on the ABR results form the operating room. The degrees and the types of hearing loss also documented in most of the cases. In addition, multiple factors that may affect the outcomes of ABR in the operating room identified. *Conclusions*. Hearing loss in children with complicated medical issues can be accurately evaluated via ABR testing in the operating room. Efforts should be made to eliminate adverse factors to ABR recording, and caution should be taken when interpreting ABR results from the operating room.

## 1. Introduction

About 2 to 3 of every 1000 children are identified with hearing loss at birth each year in the United States, and hearing impairment is, in fact, the most common sensory deficit in the pediatric population [1–4]. Late-onset hearing loss or acquired hearing loss, in addition to congenital hearing loss, is prevalent as well in young children. For example, hearing loss associated with otitis media with effusion (OME) can be seen in 15–40% of children under 5 years [5]. Since hearing impairment in early childhood can cause significant delays in speech/language developments, early identification and diagnosis of hearing loss become the initial and a critical step for proper treatment and habilitation, regardless of the etiology or the severity of the hearing loss. In clinical audiology, behavioral hearing evaluation is considered the “gold standard” for evaluating hearing sensitivity in the pediatric population. During a hearing evaluation, audiologists typically use visual reinforcement audiometry (VRA), conditioned play audiometry (CPA), or conventional pure-tone audiometry to test children's hearing. The technique chosen by an audiologist for a specific child is usually dependent upon the child's age and the child's developmental skill level. Due to developmental and physical limitations, behavioral hearing test such as VRA is not possible for any young children under six months of age. Therefore, an electrophysiology-based hearing evaluation such as the Auditory Brainstem Responses (ABR) test becomes the obvious choice for this population. It can be very difficult, if not impossible, for audiologists to conduct behavioral hearing assessments on children with complicated medical conditions and those who are unable to cooperate for the hearing test. In such cases, ABR test is frequently chosen as the method to estimate hearing thresholds. Occasionally, ABR test may be requested by physicians to be performed in the operating room in conjunction with other surgical procedures, for which patients are put under general anesthesia. 

While studies have shown that ABR test is a reliable and an objective method to assess children's hearing sensitivity [6–10], the accuracy of ABR results obtained in the operating room has been questioned in previous clinical studies since the ABR outcomes can be affected by many factors in the operating room. Several studies in the past few years suggested that ABR results, obtained in the operating room following otologic procedures such as myringotomy and tube placement, can be inaccurate, overestimating the hearing loss in children who have OME [11–13]. To better understand the benefits and limitations of performing ABR tests in the operating room, we conducted a comprehensive review of all ABR tests conducted in the operating room at Children's Hospital Boston from 2007 to 2010. In doing so, we sought to identify factors that may have some effects on the outcomes of ABR test. We also would like to share our clinical experience of performing ABR testing in the operating room.

## 2. Materials and Methods

A retrospective study, approved by the Institutional Review Board at Children's Hospital Boston, was conducted on children who had ABR test completed in the operating room in a pediatric tertiary care facility from 2007 to 2010. 

### 2.1. Patient Selection

Our database included 452 ABR tests completed in the operating room during the study period. A total of 267 pediatric cases, most of them young children, were selected for a thorough review and analysis. All selected cases had sufficient information in their medical records, which include ABR results from the operating room, medical and surgical procedure notes, and follow-up evaluation documents. 

### 2.2. Audiologic Evaluation

All audiological evaluations were carried out by licensed audiologists. ABR testing in the operating room was performed after ear examination under the microscope by a pediatric otolaryngologist while patients were under general anesthesia. Cerumen was removed when present before ABR testing. Other otologic procedures, when deemed necessary, were also performed by attending physicians/surgeons before ABR testing. These procedures included myringotomy, suction of middle ear effusion, and tube placement when necessary. The ABR was recorded with Bio-logic Navigator Pro Evoked Potential system (Natus Medical Inc., San Carlos, CA) using Children's Hospital Boston pediatric protocol. Specifically, stimuli (clicks and tonebursts), calibrated in dB normal hearing level, were presented at 38.1 per second via insert phones. Electroencephalographic signals were collected via surface electrodes and averaged. Thresholds of ABR were determined by the lowest intensity level at which the wave V was present. If the wave V of ABR was not identified with the highest intensity stimulus, extra recordings with both rarefaction and condensation clicks were made to investigate the presence/absence of the cochlear microphonics. Follow-up audiological evaluation was conducted during return visit when patients recovered from the surgical procedures, usually within 3 months. Age-appropriate behavioral test procedures, for example, VRA, CPA, or conventional pure-tone audiometry, were typically performed. In some cases, a repeat of ABR testing was conducted due to inability to complete behavioral tests or lack of cooperation from the patients. For each patient, frequency-specific hearing thresholds were documented and stored for future analysis. 

### 2.3. Data Analysis

Both descriptive and qualitative analysis was performed in this study. For all patients, clinical diagnosis, the types of hearing loss, and the degree of hearing loss, so forth were summarized. We were able to collect hearing results from ABR completed in the operating room and postoperative examination from 121 children with OME, and comparison analysis was conducted for this subgroup of patients. The mean of thresholds at three frequencies, 1000, 2000, and 4000 Hz, was calculated and defined as the averaged hearing threshold. Analysis of variance (ANOVA) and nonparametric testing (Mann-Whitney *U* test) was carried out using the Statistical Package for Social Science (SPSS v.16.0, SPSS Inc., Chicago, IL) software. 

## 3. Results

### 3.1. Patient Characteristics

Among the 267 patients included in this study, there were 156 boys and 111 girls, with age ranging from 2 months to 18 years. The request for an ABR test in the operating room was made by patients' attending physicians or managing audiologists due to previous unsuccessful attempt of hearing evaluation and patients' medical condition being not suitable for further testing in the clinic. Most of the ABR tests were carried out in the operating room while patients were under general anesthesia for other medical procedures as well. The ABR test was performed primarily to address the concern of hearing loss. There were a variety of specific reasons for clinicians and parents to question patients' hearing ability, summarized in Table 1. The majority (57.3%) of patients had recurrent otitis media, and most of them needed surgical intervention, such as myringotomy and tube placement. In these cases, ABR testing was completed after the surgical procedures to determine their hearing status. There were 36 patients (13.5% of all cases) with at least one type of the neurological disorder, who needed the ABR test in the operating room to find out their hearing ability. These neurological disorders included, but were not limited to, seizures, cerebral palsy, hydrocephalus, brain hemorrhage, ischemic encephalopathy, Graves disease, Chiari malformation, and so forth. There were 29 patients, accounted for 10.9% of all cases, who had chromosomal anomalies (e.g., Trisomy 21 or Trisomy 18, Fragile X, and other chromosomal deletions and/or duplications) and related medical issues. Sixteen patients with autistic spectrum disorders, such as autism and pervasive developmental disorders-not otherwise specified (PDD-NOS) needed the ABR test to find out if their hearing was adequate for communication. Fifteen patients with infectious diseases involving their central nerve system, such as meningitis and cytomegalovirus (CMV) infection, had ABR test in the operating room to see if their auditory function was compromised. Eleven children with the diagnosis of global developmental delay (with not-yet-identified causes) needed the ABR test to address the question whether they were able to hear adequately for verbal communication. Moreover, there were four patients with head injury and temporal bone fracture who underwent ABR test in the operating room to determine if hearing loss was involved. At last, 3 young children with known hearing loss underwent ABR testing in the operating room to confirm the degree of hearing loss before cochlear implantation. 

### 3.2. ABR Results

Based on the ABR results, we were able to determine the degree of the hearing loss for each patient. Patients' hearing was categorized according to their averaged hearing thresholds. Specifically, we defined the averaged hearing threshold better than 25 dB as normal hearing; the averaged hearing threshold between 25 dB and 40 dB as mild hearing loss; the averaged hearing threshold between 41 dB and 60 dB as moderate hearing loss; the averaged hearing threshold 61 dB and 90 dB as severe hearing loss; the averaged hearing threshold higher than 90 dB as profound loss. Our results found 58 patients (21.7%) had normal hearing. Among the other 209 children with hearing loss, 94 patients (35.2%) were found with mild loss, 75 patients (28.1%) with moderate loss, 21 patients (7.9%) with severe loss, and 19 patients (7.1%) with profound loss. In addition, we were able to identify the types of hearing loss in most of our patients. Over 60% of the children identified with hearing loss had conductive loss while about 15% of them had sensorineural loss. In addition, mixed hearing loss was seen in 17 patients (8.1%). There were 8 patients whose hearing loss was consistent with auditory neuropathy spectrum disorders. Based on the available results, the type of hearing loss could not be determined for the remaining 26 patients. The above findings are summarized in Tables 2 and 3, respectively. 

### 3.3. Identifiable Medical Conditions or Events Associated with Hearing Loss

By reviewing patients' medical records, we identified a number of medical conditions or events that are known to be associated with hearing loss, as summarized in Table 4. Over 50% (109 patients) of the patients with hearing loss were found having OME as a major contributing cause for hearing losses. There were 21 patients (about 10%) with syndromic diseases such as CHARGE syndrome and Waardenburg syndrome, in which hearing loss is common. Congenital CMV and meningitis were diagnosed in 13 patients with hearing loss while metabolic diseases were seen in 9 children with hearing loss. There were 9 patients with hearing loss who had a history of chemotherapy and/or antibiotics treatments, which were known to be ototoxic and can cause significant hearing loss. Moreover, 9 children with hearing loss previously underwent extracorporeal membrane oxygenation (ECMO) treatment. Inner ear structural anomalies in the temporal bone, such as enlarged vestibular aqueduct and malformed cochlea, were found in 9 patients with hearing loss. Other medical issues, such as neonatal hypoxia, prematurity, and hyperbilirubinemia, were noted in the remaining 33 patients with documented hearing loss. 

### 3.4. Possible Factors Affecting the ABR Outcomes in the Operating Room

During our recordings of ABR in the operating room, we observed several factors that could affect the outcomes. First, the background noise level in the operating room, in some cases, was extraordinarily high due to the use of multiple medical devices, which could create elevated ABR thresholds at lower frequencies such as 500 and 1000 Hz. Second, electromagnetic interferences from power sources, medical equipment in the operating room, and patients' own medical devices (such as pacemaker and vagal nerve stimulator) could degrade ABR responses, which made the ABR waveforms unclear and difficult to read, resulting in overestimation of hearing loss. Third, the presence of middle ear fluid could influence the hearing results from the ABR testing even if the fluid was removed surgically, more significant in the cases with mucoid than in the cases with serous effusion. Finally, the ABR could be affected by patients' physiological and neurological status which might be altered by anesthesia medications. These factors are summarized in Table 5.

### 3.5. Comparison Analysis

Since the majority of our patients had OME, we decided to do further analysis on this group to see if there were any other factors of significance. Among the 153 children with suspected OME, we were able to collect pre- and postoperative hearing results in 121 patients. We divided them into three groups based on the procedures they received in the operating room. Group 1 included 47 patients (69 ears) who underwent microscopic examination with no middle ear effusion found; Group 2 included 13 patients (18 ears) who underwent myringotomy only with no tube placement; Group 3 included 61 patients (83 ears) who underwent myringotomy and tube placement. For patients in Group 1, the averaged hearing threshold from ABR was 3.5 dB (±3.5) higher than the follow-up audiological evaluation. In Group 2, the averaged hearing threshold from ABR was 4.0 dB (±3.5) higher than the follow-up audiological evaluation. In Group 3, the averaged hearing threshold of ABR from the operating room was 9.4 dB (±10.6) higher than the follow-up audiological evaluation. Comparison analysis showed that the averaged hearing threshold discrepancy between ABR from the operating room and the follow-up audiological evaluation was significantly different among the three groups (*P* < 0.01). 

Although the averaged hearing threshold discrepancies between the ABR from the operating room and the follow-up audiological evaluation seemed to be relatively small in all groups, the results varied greatly among patients. Across the three groups, there were 16 ears that had average hearing threshold difference of greater than 20 dB, with the majority of these ears being patients in Group 3. In all groups, threshold discrepancy between the ABR from the operating room and the follow-up audiological evaluation was notably higher at 1000 Hz than that at 2000 and 4000 Hz. 

## 4. Discussion

One common goal in identification of hearing loss in children among hearing care professionals, including otolaryngologists and audiologists, is making an accurate diagnosis as early as possible so that children with hearing loss can receive appropriate treatment and habilitation to avoid delays in speech and language development. Clinically, audiologists will choose age-appropriate behavioral evaluation methods to obtain hearing thresholds. ABR test may be used in certain cases for difficult-to-test children to estimate their hearing sensitivity although its value remains debatable among professionals when it is used in the operating room. 

Our experience of performing ABR testing in the operating room, as shown in our study, is mostly successful, and our findings are tremendously valuable in clinical care of pediatric patients with hearing concerns. Not only are we able to define the hearing status for the most difficult-to-test children with a variety of serious medical issues, but we are also able to determine the degree and the type of hearing loss for the majority of the patients. By doing so, we have provided appropriate recommendations for their much needed treatment and habilitation. At the same time, we also gained more knowledge of medical conditions and events that are linked to hearing loss. 

While we are confident in the justification of using ABR in the operating room [14, 15], we remain aware of the recent clinical studies which have revealed significant discrepancies between ABR results obtained in the operating room and the results obtained in the follow-up period. These studies have raised a concern regarding the validity of ABR outcomes obtained in the operating room [11–13]. Therefore, we systematically investigated the ABR results from the operating room and follow-up audiologic evaluation in a large group of cases. We found that hearing thresholds obtained from the ABR tests in the operating room were significantly elevated in patients who underwent myringotomy plus tube placement. On the other hand, minimal discrepancies, smaller than 5 dB, were found in the majority of patients without middle ear effusion and surgical procedures involved. Our findings also suggest that the elevated ABR thresholds in the operating room are most often temporary and usually resolve within a short time period after their surgery. Mechanisms of the temporary shift in hearing may include (1) the existence of residual fluid in the middle ear space following the surgical procedures; (2) the high-intensity noises created by suction, which could cause a noise-induced temporary threshold shift in hearing [11, 16]; (3) swelling and inflammation of the membranes in the middle ear created by suctioning of fluid and insertion of ear tubes. 

Performing ABR testing in the operating room can be, at times, a challenge. Through the process of conducting hundreds of ABR tests in the operating room, our audiology staff has experienced their success and difficulties in this task and our best practice have evolved. Efficiency of troubleshooting adverse ABR recording conditions, for example, eliminating electromagnetic inferences and minimizing factors or events that may degrade ABR waveform, has improved among our audiologists. ABR testing in the operating room, unlike the behavioral hearing tests, is not a direct measure of patients' hearing sensitivity. Accordingly, audiologists have to adjust their routine in explaining the audiologic results, improve their counseling skills, and provide more appropriate recommendations regarding patients' hearing loss to their parents. At last, better coordination and communication with other medical professionals in the operating room, such as the nurses, the surgeons, and anesthesiologists, is a key for audiologists to achieve the goal of accurate hearing assessment for children with complicated medial issues. 

## 5. Conclusion

Based on our study, we have concluded that ABR testing can be successfully performed in the operating room when interference factors are properly addressed. In fact, hearing thresholds can be accurately estimated based on the outcomes of ABR conducted in the operating room under the right circumstances, for example, in the absence of middle ear effusion or surgical procedure involved. In addition, the degrees and the types of hearing loss can be determined in most of the cases. When myringotomy and tube placement are done prior to the ABR testing, follow-up hearing evaluation when the ear is cleared and healthy, either by behavioral tests or by ABR testing, is necessary to determine a patient's true hearing thresholds or to confirm the hearing loss established by the ABR results from the operating room. Caution should be taken when interpreting the ABR results from the operating room, and appropriate parental counseling must be provided. It has to be noted that there are times in which ABR test in the operating room is the only option to assess a child's hearing ability.

## Figures and Tables

**Table 1:** A summary of specific reasons for ABR testing in the operating room, with the number of cases in each category listed.

Specific reasons for ABR test	Number of cases	Percentage
Recurrent otitis media	153	57.3%
Neurologic disorders	36	13.5%
Chromosomal anomalies	29	10.9%
Autistic spectrum disorders	16	6.0%
Infectious disease involving central nerve system	15	5.6%
Global developmental delay	11	4.1%
Head injury/temporal bone fracture	4	1.5%
Verification of hearing loss for cochlear implant	3	1.1%

Total	267	100%

**Table 2:** The distribution of hearing outcomes based on the ABR test in the operating room.

Degrees of hearing loss	Number of cases	Percentage
Normal hearing (<25 dB)	58	21.7%
Mild loss (25–40 dB)	94	35.2%
Moderate loss (41–60 dB)	75	28.1%
Severe loss (61–90 dB)	21	7.9%
Profound loss (>90 dB)	19	7.1%

Total	267	100%

**Table 3:** Different types of hearing loss found in 209 patients.

Types of hearing loss	Number of cases	Percentage
Conductive	126	60.3%
Sensorineural	32	15.3%
Mixed	17	8.1%
Auditory Neuropathy Spectrum Disorders	8	3.9%
Undetermined	26	12.4%

Total	209	100%

**Table 4:** A list of medical conditions events contributing to hearing loss. OME: otitis media with effusion; ECMO: extracorporeal membrane oxygenation treatment; CMV: cytomegalovirus infection.

Medical conditions/events associated with hearing loss	Number of cases	Percentage
OME	109	52.2%
Syndromic diseases	21	10.1%
CMV and bacterial meningitis	13	6.2%
Metabolic diseases	9	4.3%
Ototoxicity	9	4.3%
ECMO	8	3.8%
Temporal bone/inner ear structural anomalies	7	3.3%
Others	33	15.8%

Total	209	100%

**Table 5:** Potential factors affecting ABR outcomes in the operating room.

		Potential effects on ABR
Surgical-related factors	Surgical procedures	Temporary shift in thresholds
	Middle ear effusion	Elevation of thresholds
	Use of anesthesia medication	Poor ABR recording

Equipment-related factors	Power source	60 Hz interference on ABR recording
	Medical devices used in the operating room	Electromagnetic interferences
	Patients' own medical devices	Electromagnetic interferences
	High level background noises	Elevation of low-frequency ABR thresholds

Patients' physiologic and neurologic status	Changes in blood oxygen level, blood pressures and body temperature, and so forth.	Poor ABR waveforms, delays in ABR latencies, and reduction of ABR amplitude.
